# Complementary role of echocardiography, karyotyping, and chromosomal microarray in congenital cardiac anomalies

**DOI:** 10.3389/fmed.2025.1586161

**Published:** 2025-08-29

**Authors:** Jun Yin, Xiaomeng Zhang, Qingsong Wang, Qian Cao, Huimin Ou, Zhihui Zhao, Shuqiong Xu, Junru Wang, Li Xia, Bin Zhang, Xuemei Xiao, Tongyong Luo, Xianmin Wang

**Affiliations:** ^1^Department of Ultrasound, Jintang First People’s Hospital, West China Hospital Sichuan University Jintang Hospital, Chengdu, Sichuan, China; ^2^Pediatric Cardiology Center, Sichuan Provincial Women’s and Children’s Hospital, The Affiliated Women’s and Children’s Hospital of Chengdu Medical College, Chengdu, Sichuan, China; ^3^Department of Pediatrics, Jintang First People’s Hospital, West China Hospital Sichuan University Jintang Hospital, Chengdu, Sichuan, China; ^4^Department of Radiology, Sichuan Provincial Women’s and Children’s Hospital, The Affiliated Women’s and Children’s Hospital of Chengdu Medical College, Chengdu, Sichuan, China; ^5^Department of Neonatology, Sichuan Provincial Women’s and Children’s Hospital, The Affiliated Women’s and Children’s Hospital of Chengdu Medical College, Chengdu, Sichuan, China

**Keywords:** congenital cardiac anomalies, echocardiography, chromosome karyotyping, chromosomal microarray, prenatal diagnosis, genetic testing

## Abstract

**Objective:**

To assess the diagnostic efficacy of echocardiography, chromosome karyotyping, and chromosomal microarray analysis (CMA) in congenital cardiac anomalies.

**Methods:**

This retrospective cohort study analyzed data from 3,386 pregnant women who underwent echocardiography and amniocentesis at the Sichuan Provincial Maternal and Child Health Care Hospital between January 2020 and August 2022. The study group included 697 women whose fetuses were diagnosed with congenital heart disease (CHD) by echocardiography, while the comparison group included 2,689 women with normal echocardiographic results. The diagnostic contributions of echocardiography, karyotyping, and CMA were compared between the two groups.

**Results:**

Among the 697 cases diagnosed with CHD, the most common types were ventricular septal defect (44.45%) and valve abnormalities (40.66%). Chromosomal abnormalities were detected in 41 out of 629 CHD cases (6.52%) by karyotyping, with higher rates in complex CHD (16.36%) and CHD with extracardiac anomalies (23.08%) compared to the comparison group (4.71%). CMA identified 34 pathogenic copy number variations (CNVs) (5.28%) and 9 variants of unknown significance (VOUS) (1.40%) in 644 CHD cases, with higher CNV detection rates in complex CHD (7.69%) and CHD with extracardiac anomalies (7.69%) compared to the comparison group (1.38%). CMA further identified pathogenic CNVs in 4.42% (26/588) of CHD cases with a normal karyotype, yielding an incremental diagnostic rate of 4.42%.

**Conclusion:**

Echocardiography remains the cornerstone for the prenatal detection of fetal heart malformations. When combined with karyotyping and CMA, this integrated approach achieves maximal detection of both macroscopic and submicroscopic genomic alterations—particularly in complex cardiac malformations or when extracardiac anomalies coexist—thereby delivering timely, comprehensive genetic information to guide early intervention and tailored perinatal counseling.

## 1 Introduction

Congenital heart disease (CHD) is among the most prevalent congenital structural anomalies, with a global incidence ranging from 0.8 to 1.2% (1). Alarmingly, the prevalence of CHD is on the rise, presenting significant challenges to perinatal care and long-term pediatric management. CHD can be broadly categorized into simple and complex types based on the anatomical complexity of the cardiac malformations. Simple CHD, which includes conditions such as ventricular septal defects and mild valve abnormalities, is more common and generally associated with a favorable prognosis. In contrast, complex CHD, encompassing conditions like tetralogy of Fallot and transposition of the great arteries, accounts for less than 20% of all cases but is often associated with significant hemodynamic changes and severe neonatal outcomes, including stillbirth, neonatal disability, or death (2). Given the profound impact of complex CHD on the quality of life and long-term prognosis of affected children, early and accurate diagnosis is crucial for effective clinical intervention and improved outcomes.

Advancements in medical imaging technologies have significantly enhanced the prenatal diagnosis of fetal cardiac malformations. Ultrasound, particularly fetal echocardiography, serves as the primary tool for prenatal screening, providing real-time imaging of fetal heart morphology, structure, and hemodynamics (3). This modality is invaluable for the early detection of structural malformations and guiding both prenatal and postnatal management strategies, thereby leading to notable improvements in clinical outcomes and treatment effectiveness. Despite its critical role, echocardiography remains the gold standard for the prenatal diagnosis of CHD, although it has limitations in detecting certain genetic defects (4).

With increasing recognition of the role of chromosomal abnormalities and genetic factors in fetal cardiac malformations, genetic testing has emerged as a valuable adjunct for the early identification of CHD (5). Genetic testing, including chromosome karyotyping and chromosomal microarray analysis (CMA), provides crucial complementary information, especially for prognosis and counseling, particularly when genetic syndromes are involved. While genetic findings may influence clinical management strategies, they do not replace echocardiography in the diagnosis of structural cardiac defects.

This study aims to evaluate the diagnostic efficacy of echocardiography, chromosome karyotyping, and CMA in the prenatal diagnosis of congenital cardiac anomalies. By analyzing the strengths and limitations of these three diagnostic modalities, we seek to elucidate their contributions to the early detection of fetal cardiac malformations. We hope this study will provide precise strategies for early diagnosis, optimize prenatal screening, enhance diagnostic accuracy, and offer scientific support for interventions and prognostic guidance in fetal health.

## 2 Materials and methods

### 2.1 Study population

This study employed a retrospective analysis, collecting data from 3,386 pregnant women who underwent invasive amniocentesis due to various high-risk factors between January 2020 and August 2022 at the Sichuan Provincial Maternal and Child Health Care Hospital, Affiliated Women’s and Children’s Hospital of Chengdu Medical College. These high-risk factors included advanced maternal age (≥35 years), adverse obstetric history, high-risk results from non-invasive prenatal testing (NIPT) for trisomy 21, family history of genetic disorders, and others. The women were examined during early (11–13+6 weeks) and/or mid-pregnancy (20–28 weeks). Among them, 697 women whose fetuses were diagnosed with cardiac abnormalities via fetal echocardiography, including those with or without extracardiac anomalies, were classified as the study group. The inclusion criteria for the study group were: (1) diagnosis of fetal cardiac abnormalities, including those with or without extracardiac anomalies, by two qualified physicians; (2) consent for invasive prenatal diagnosis following genetic counseling and signing of the relevant informed consent forms. The exclusion criteria were: (1) presence of explicit contraindications for invasive prenatal diagnosis, or refusal of invasive prenatal diagnosis after counseling; (2) fetal cardiac phenotypes involving only arrhythmia or pericardial effusion. The control group included 2,689 pregnant women who underwent amniocentesis and completed karyotype analysis and/or copy number variation analysis, but whose fetuses had no developmental abnormalities detected by ultrasound. To ensure the comparability of the study group and control group, we compared the distribution of high-risk indicators between the two groups. The results showed that there were no statistically significant differences in the distribution of high-risk factors between the study group and the control group (*P* > 0.05 for all comparisons), indicating that the two groups were comparable in terms of baseline characteristics, as shown in Table 1.

**TABLE 1:** Distribution of high-risk factors between the study group and control group.

High-risk factor	Study group (*n* = 697)	Control group (*n* = 2689)	χ^2^ value	*P*-value
Advanced maternal age (≥35 years)	244 (35.0%)	878 (32.6%)	1.92	0.166
NIPT high risk (Trisomy 21)	107 (15.4%)	439 (16.3%)	0.52	0.471
Adverse obstetric history	146 (20.9%)	519 (19.3%)	1.32	0.251
Family history of genetic disorders	85 (12.2%)	352 (13.1%)	0.56	0.454
Other high-risk factors*	71 (10.2%)	296 (11.0%)	0.52	0.471
Two or more high-risk factors	44 (6.3%)	205 (7.6%)	2.05	0.152

*“Other” includes high-risk factors not listed separately, such as multiple pregnancies, risk of intrauterine infection, abnormal metabolic disease screening, and exposure to teratogenic factors in early pregnancy.

### 2.2 Criteria for the diagnosis and classification of cardiac anomalies

In this study, the selection and classification of cardiac anomalies were based on the diagnostic criteria for CHD established by the Ministry of Health of China. The criteria for diagnosing and classifying CHD are as follows:

(1) Diagnosis of CHD: Fetal echocardiography was used as the primary tool for diagnosing CHD. All echocardiograms were performed by two qualified physicians, and the final diagnosis was confirmed by consensus. The diagnostic criteria included the identification of structural abnormalities in the fetal heart, such as septal defects, valve abnormalities, and other cardiac malformations.
(2) Classification of CHD: CHD was categorized into three subgroups according to the diagnostic criteria for birth defects set by the Ministry of Health of China: ➀Simple CHD: Isolated intracardiac lesions that generally do not cause significant hemodynamic changes, such as isolated ventricular septal defects, mild aortic valve stenosis, and mild pulmonary valve stenosis. ➁ Complex CHD: The coexistence of one or more intracardiac and great vessel structural malformations with significant hemodynamic changes, including tetralogy of Fallot, transposition of the great arteries, single ventricle, double outlet right ventricle, complete atrioventricular septal defect, truncus arteriosus, pulmonary atresia, total anomalous pulmonary venous return, hypoplastic left heart syndrome, interrupted aortic arch, aortic coarctation, and heterotaxy syndrome. ➂ CHD with extracardiac anomalies: Cases involving anomalies in systems other than the cardiovascular system, such as absent fetal nasal bone, increased nuchal translucency, esophageal atresia, choroid plexus cysts, and talipes equinovarus.

In the statistical analysis, cases with one or more types of complex CHD were counted as a single case of complex CHD. Cases with multiple simple CHD types occurring simultaneously were categorized as simple CHD, as they do not meet the criteria for complex CHD (i.e., they do not cause significant hemodynamic changes). This method ensures accurate classification based on the most severe pathological features present in each case. In cases where complex CHD coexists with extracardiac anomalies, the case is classified solely under the category of “CHD with extracardiac anomalies” to avoid duplicate counting and to reflect the most severe phenotypic presentation in the analysis.

### 2.3 Ultrasound examination

All pregnant women underwent ultrasound echocardiography using a GE Voluson E8 color Doppler ultrasound diagnostic system, with a probe frequency of 4–8 MHz. Using a transabdominal volumetric probe, the physician first located the intrauterine position of the fetus and the fetal heart. Subsequently, the heart was examined in detail using various views, including the four-chamber view, left and right ventricular outflow tract views, and vascular views. The size, shape, and structure of the cardiac chambers, the continuity of the atrial septum, the presence of blood flow shunts, and the development, position, diameter, and motion of the major vessels and valves were carefully observed. The examinations were independently performed by two prenatal diagnostic-qualified physicians, and the final diagnosis was confirmed by consensus between the two physicians.

In addition to the detailed cardiac examination, the position and structure of the fetal heart were assessed to determine the situs. Situs was categorized as normal (situs solitus), reversed (situs inversus), or ambiguous (situs ambiguous). Any anomalies detected were recorded and included in the final diagnosis.

Due to resource constraints and clinical practice, systematic postnatal diagnostic confirmation was not performed for all neonates. However, all fetuses underwent at least two prenatal echocardiograms to ensure the accuracy of the prenatal diagnosis.

### 2.4 Amniocentesis for chromosomal karyotyping

Amniocentesis was performed under ultrasound guidance. The procedure involved first determining the accurate position of the fetus and fetal heart under B-mode ultrasound, followed by local anesthesia at a suitable puncture site. A 10–20 mL sample of amniotic fluid was aspirated and centrifuged. The sediment was inoculated into culture medium (BIO-AMF-2) and cultured for 7 days. After cell collection, hypoosmotic treatment, and fixation, chromosomal karyotyping was performed using G-banding techniques according to the international standard (ISCN2013). All fetuses underwent chromosomal karyotyping, and CMA was performed when necessary.

### 2.5 Chromosomal microarray analysis

Chromosomal microarray analysis was conducted using the Agilent aCGH DNA Labeling Kit to detect copy number variations (CNVs) and loss of heterozygosity (LOH) in genomic DNA. The experimental procedures followed the product instructions, and data analysis was performed using the CytoGenomics and Gennoglyphix software. Results were interpreted by comparing multiple databases (such as DECIPHER, ClinVar, DGV, etc.). All CNV results were classified according to the Medical Genetics and Genomics (ACMG) guidelines into pathogenic CNVs, VOUS, and benign CNVs. Final reports were provided by qualified physicians, and detailed descriptions of any potential CNVs associated with cardiac malformations were included. Any CNV classified as a VOUS was re-analysed using the identical CMA platform in parental samples whenever available to establish inheritance.

To ensure temporal consistency across the cohort, we analysed the 3,386 samples between August and October 2024 under the 2020 ACMG/ClinGen CNV interpretation standards, including the 2023 sequence-variant amendments. Reference databases (ClinVar, DECIPHER, DGV, OMIM) were frozen at their August 2024 releases and used as the sole knowledge base. Two board-certified clinical geneticists independently reviewed each CNV call, resolving discrepancies by consensus. This protocol guarantees that every case acquired from January 2020 to August 2022 was interpreted within an identical guideline–database framework, thereby eliminating batch effects. Continuous surveillance from October 2024 through May 2025 has identified no updates to the guidelines or databases that would affect the interpretation of fetal CHD-related CNVs.

### 2.6 Follow-up

All study participants were followed up after delivery, with a focus on the clinical outcomes of fetal cardiac malformations. Low-risk pregnant women were followed up for 3–6 months post-delivery, while high-risk pregnant women, particularly those with clinically significant CNVs, were followed for an extended period to ensure comprehensive monitoring of fetal development and clinical consequences. To validate the negative predictive value of prenatal screening, we consecutively and unselectively enrolled the first 400 neonates from the control group for universal postnatal echocardiography within 72 h of birth; 368 (92.0%) completed the scan, and none were referred because of clinical signs.

### 2.7 Statistical analysis

This study used a retrospective design, categorizing and organizing the collected data, which was presented as count data. Statistical analysis was performed using SPSS version 25.0. The chi-square test (Fisher’s exact test) was used to analyze the differences in chromosomal abnormalities and the pathogenic detection rate of CNVs between the groups. A significance level of α = 0.05 was set, and differences with *P* < 0.05 were considered statistically significant.

## 3 Results

### 3.1 Ultrasound examination results

A total of 3386 pregnant women underwent fetal heart examinations using ultrasound imaging. Among them, 697 cases were identified with CHD. Based on the type of cardiac defect, these 697 CHD cases were classified into 9 categories (see Table 2). The most common defect was isolated septal defects, accounting for approximately 44.45%, with ventricular septal defects being the most prevalent. This was followed by isolated valve abnormalities, including valvular regurgitation, stenosis, and atresia, which accounted for about 40.66%. Isolated conus arteriosus anomalies, including Tetralogy of Fallot, and situs anomalies were the least common, accounting for approximately 0.24 and 0.39%, respectively. Based on the diagnostic criteria for birth defects set by the Ministry of Health of China and the severity of CHD, the cases were broadly categorized into 3 groups: simple CHD (602/697, 86.37%), complex CHD (69/697, 9.9%), and CHD with extracardiac anomalies (26/697, 3.73%). Simple CHD and complex CHD were further combined as independent CHD types.

**TABLE 2:** Classification of CHD and ultrasound diagnosis results.

No.	CHD type	Ultrasound diagnosis results	Percentage (%)
A	Septal defects	Ventricular septal defect, atrial septal defect	44.54
B	Isolated valve abnormalities	Mitral valve regurgitation, tricuspid valve regurgitation, aortic valve regurgitation, pulmonary valve regurgitation	40.66
C	Right ventricular outflow tract abnormalities	Tricuspid stenosis, tricuspid atresia, pulmonary valve atresia, etc.	3.12
D	Left ventricular outflow tract abnormalities	Mitral valve stenosis, aortic valve stenosis, interrupted aortic arch, etc.	1.29
E	Isolated conus arteriosus abnormalities	Tetralogy of Fallot, complete transposition of the great arteries, persistent arterial duct	0.24
F	Complex conus arteriosus abnormalities	Phenotype from group E with additional intracardiac anomalies	0.81
G	Left ventricular hypoplasia syndrome	Left ventricular hypoplasia syndrome	7.9
H	Situs anomalies syndrome	Situs anomalies syndrome	0.39
I	Others:	Complete pulmonary venous return anomaly, single ventricle, single atrium, persistent left superior vena cava, etc.	1.05

### 3.2 Karyotype analysis results

Among the 697 cases of CHD, 66 cases were excluded due to lack of karyotype analysis and 2 cases failed to culture. A total of 629 cases underwent chromosomal karyotype analysis, and chromosomal abnormalities were detected in 41 cases (41/629, 6.52%). Among these, 14 cases (14/629, 2.23%) exhibited chromosomal number variations, including 2 cases of Trisomy 21, 7 cases of Trisomy 18, 2 cases of sex chromosome duplications, and 3 cases of mosaicism. A total of 27 cases (27/629, 4.29%) showed chromosomal structural abnormalities, including duplications, deletions, inversions, translocations, and insertions, as detailed in Table 3.

**TABLE 3:** Karyotype results by CHD subtype.

CHD subtype	N karyotyped	Normal *n* (%)	Abnormal *n* (%)	Culture failure *n*	Abnormal rate %
Simple CHD	550	523 (95.1)	26 (4.7)	1	4.73
Complex CHD	55	46 (83.6)	9 (16.4)	0	16.36
CHD with extracardiac abnormalities	26	19 (73.1)	6 (23.1)	1	23.08
Total CHD	631	588 (93.2)	41 (6.5)	2	6.52

In the experimental group, 550 cases of simple CHD underwent karyotype analysis, with 1 case of failed culture. Among the remaining 549 cases, 523 had a normal karyotype, while 26 exhibited chromosomal abnormalities, yielding a karyotype abnormality detection rate of 4.73%. In the complex CHD group, 55 cases underwent karyotype analysis, including 46 cases with a normal karyotype and 9 cases with abnormal karyotypes, resulting in an abnormality detection rate of 16.34%. In the CHD group with extracardiac abnormalities, 19 cases were found to have a normal karyotype, and 6 had abnormal karyotypes, with a karyotype abnormality detection rate of 23.08%, excluding 1 case of culture failure. In the control group, a total of 2208 cases underwent karyotype analysis, with 4 failed cultures. Of the remaining 2204 cases, 2100 had normal karyotypes, and 104 had abnormal karyotypes, resulting in a chromosomal abnormality detection rate of 4.71%. Comparison of the four groups revealed that the control group had the lowest karyotype abnormality detection rate (4.71%), while the CHD group with extracardiac abnormalities had the highest rate (23.08%). All cases with chromosomal numerical abnormalities showed cardiac abnormalities combined with multiple extracardiac malformations. When comparing the three experimental groups with the control group, the differences in karyotype abnormality detection rates between the simple CHD, complex CHD, and CHD with extracardiac abnormalities groups were statistically significant (*P* < 0.05), with the detection rates higher than those in the control group (see Table 4).

**TABLE 4:** Comparison of karyotype results between experimental and control groups.

Group	Normal karyotype	Abnormal karyotype	Culture failure	Total	Abnormal karyotype positive rate	*P*-value
Simple CHD	523	26	1	550	4.73%	0.032
Complex CHD	46	9	0	55	16.36%	0.014
CHD with extracardiac abnormalities	19	6	1	26	23.08%	0.01
Control group	2100	104	4	2208	4.71%	

### 3.3 CNV analysis results

#### 3.3.1 Comparison of CNVs between experimental and control groups

In 697 cases of CHD, excluding 53 cases that did not undergo CNVs analysis, 644 cases underwent CNVs detection, resulting in 43 cases (43/644, 6.68%) with CNVs detected. Among these, 34 cases (34/644, 5.28%) had pathogenic CNVs, and 9 cases (9/644, 1.40%) had VOUS. Compared to traditional chromosomal karyotyping, the CMA method demonstrated a higher detection rate, as detailed in Table 5.

**TABLE 5:** CMA results by CHD subtype.

CHD subtype	N tested	Normal CNV *n* (%)	Pathogenic CNV *n* (%)	VOUS *n* (%)	Pathogenic rate %
Simple CHD	553	520 (94.0)	27 (4.9)	6 (1.1)	4.88
Complex CHD	65	59 (90.8)	5 (7.7)	1 (1.5)	7.69
CHD with extracardiac abnormalities	26	22 (84.6)	2 (7.7)	2 (7.7)	7.69
Total CHD	644	601 (93.3)	34 (5.3)	9 (1.4)	5.28

Further subdivision of the experimental groups showed the following: For simple CHD, 553 cases underwent CNVs analysis, with 520 cases having normal CNVs results, 27 cases with clinically defined pathogenic or possibly pathogenic CNVs (detection rate: 4.88%), and 6 cases with VOUS (detection rate: 1.08%). For complex CHD, 65 cases underwent CNVs analysis, with 59 normal CNVs results, 5 cases with clinically defined pathogenic or possibly pathogenic CNVs (detection rate: 7.69%), and 1 case with VOUS (detection rate: 1.54%). For CHD with extracardiac abnormalities, 26 cases underwent CNVs analysis, with 22 normal CNVs results, 2 cases with clinically defined pathogenic or possibly pathogenic CNVs (detection rate: 7.69%), and 2 cases with VOUS (detection rate: 7.69%).

For the control group, 2177 cases underwent CNVs analysis without ultrasound abnormalities, with 2141 normal CNVs results, 30 cases with clinically pathogenic or possibly pathogenic CNVs (detection rate: 1.38%), and 6 cases with VOUS (detection rate: 0.28%). When comparing the three experimental groups with the control group, the control group had the lowest detection rate for pathogenic CNVs (1.38%), while the complex CHD and CHD with extracardiac abnormalities groups had the highest CNVs detection rates, both at 7.69%. The CNVs pathogenic detection rates for the experimental groups were significantly higher than that of the control group (*P* < 0.05), as shown in Table 6.

**TABLE 6:** Comparison of CNVs analysis between experimental group and control group.

Group	Normal CNVs	Pathogenic CNVs	VOUS	Total	Pathogenic or possibly pathogenic detection rate	*P*-value
Simple CHD	520	27	6	553	4.88%	<0.05
Complex CHD	59	5	1	65	7.69%	0.002
CHD with extracardiac abnormalities	22	2	2	26	7.69%	0.033
Control Group	2141	30	6	2177	1.38%	

#### 3.3.2 Distribution of pathogenic CNV segment sizes by CHD subtype

Among the 34 pathogenic CNVs identified by CMA, the segment-size distribution was as follows: in the simple-CHD group (*n* = 27), 18.5% were < 1 Mb, 48.1% were 1–4 Mb, and 33.3% were ≥5 Mb; in the complex-CHD group (*n* = 5), the proportions were 20.0%, 40.0%, and 40.0%, respectively; and in the CHD-with-extracardiac-anomalies group (*n* = 2), the corresponding values were 0%, 50.0%, and 50.0%. Overall, χ^2^ analysis revealed no significant difference in size distribution among the three groups (χ^2^ = 0.56, *P* = 0.755), as shown in Table 7.

**TABLE 7:** Distribution of pathogenic CNV segment sizes by CHD subtype.

CHD subtype	N (pathogenic CNVs)	<1 Mb *n* (%)	1–4 Mb *n* (%)	≥5 Mb *n* (%)
Simple CHD	27	5 (18.5)	13 (48.1)	9 (33.3)
Complex CHD	5	1 (20.0)	2 (40.0)	2 (40.0)
CHD with extracardiac anomalies	2	0 (0.0)	1 (50.0)	1 (50.0)

#### 3.3.3 Fetal congenital heart disease with normal chromosomal karyotype and the correlation with CNVs

Among the experimental group, there were 588 cases with a normal chromosomal karyotype. Of these, 553 cases (94.05%) showed no CNVs on copy number variation analysis, 26 cases (4.42%) had clinically confirmed or possibly pathogenic CNVs, and 9 cases (1.53%) had CNVs of unknown pathogenicity. In the control group, there were 2100 cases with a normal chromosomal karyotype. Among these, 2065 cases (98.33%) showed no CNVs on copy number variation analysis, 29 cases (1.38%) had clinically confirmed or possibly pathogenic CNVs, and 6 cases (0.29%) had CNVs of unknown pathogenicity. When comparing the experimental group with the control group in terms of CNVs detection in cases with a normal karyotype, the difference was statistically significant (*P* < 0.05). The experimental group had a higher CNVs detection rate than the control group, as shown in Table 8. Among the 26 cases in the experimental group with a normal karyotype but positive CNVs, the CNVs were located on chromosomes 1, 2, 4, 5, 7, 16, 17, 18, 20, and 22, with microdeletions and microduplications ranging from 0.017 Mb to 21.3 Mb. Of these, 24 cases had isolated congenital heart defects, and 2 cases had heart defects combined with extracardiac abnormalities.

**TABLE 8:** Comparison of CNV detection in cases with normal karyotype.

Karyotype normal group	CNV normal	CNV pathogenic/possibly pathogenic	VOUS	χ^2^	*P*-value
CHD	553 (94.05%)	26 (4.42%)	9 (1.53%)	35.486	<0.001
Control group	2065 (98.33%)	29 (1.38%)	6 (0.29%)		

### 3.4 Postpartum outcomes

Among the 697 pregnant women with fetuses diagnosed with CHD, 612 cases resulted in successful deliveries with live births (87.80%). Sixty-one cases chose elective abortion due to genetic syndromes or complex CHD, 3 cases were stillbirths, and another 21 cases opted for refusal of cephalic delivery information. The 61 aborted fetuses were primarily cases of genetic syndromic CHD and complex CHD.

We conducted postnatal echocardiography for 596 of the 612 live-born infants diagnosed prenatally with CHD, with 16 cases not undergoing postnatal echocardiography. Among these 596 cases, 591 (99.2%) had postnatal diagnoses consistent with the prenatal diagnoses. Additionally, we collected postnatal echocardiography data for 368 neonates who were not diagnosed with CHD prenatally but underwent echocardiography after birth. Among these 368 neonates, 364 (98.9%) had normal postnatal echocardiography results, confirming the absence of CHD. This comprehensive postnatal follow-up provides further validation for our prenatal diagnostic methods, with 99.2% of the cases with prenatal CHD diagnosis confirmed by postnatal echocardiography and 98.9% of the cases without prenatal CHD diagnosis confirmed to be free of CHD. These results support the accuracy of our prenatal diagnostic methods and help to assess the negative predictive value of our screening strategy.

Of the 612 live-born infants, 9 exhibited extracardiac structural abnormalities, including hypospadias, microcephaly, polydactyly, cleft lip and palate, micrognathia, external ear deformities, and unilateral renal agenesis. Among these, 1 case died shortly after birth due to metabolic abnormalities. The other newborns were in good health, with no signs of neurodevelopmental disorders or other abnormalities.

Among nine prenatally detected VOUS, trio analysis was completed in seven (77.78%): two (28.57%) were reclassified as likely benign after confirming paternal inheritance, four (57.14%) remained VOUS because of incomplete penetrance observed in the carrier parent, and one (14.29%) was upgraded to likely pathogenic on the basis of its *de novo* status and gene content.

## 4 Discussion

### 4.1 Ultrasound and genetic testing in CHD

Prenatal diagnosis plays a critical role in optimizing perinatal management, reducing neonatal mortality and associated complications, and providing essential guidance for delivery planning and genetic counseling. Fetal echocardiography, as the primary tool for prenatal detection of cardiac anomalies, is widely recognized as the gold standard for diagnosing CHD due to its high sensitivity, broad applicability, good specificity, and reproducibility (6). It can accurately detect approximately 85% of cardiac abnormalities and simultaneously assess extracardiac structural anomalies (7) . Among the various echocardiographic views, the four-chamber view (FC view) is the most fundamental and critical, offering direct visualization of cardiac morphology and serving as a key component in CHD screening (8, 9).

With the expansion of ultrasound screening from a single four-chamber view to a comprehensive six-view protocol and the continuous improvement of operator expertise, the detection rate of complex CHD has significantly increased (10). Two-dimensional ultrasound allows direct assessment of cardiac and great vessel development, and when combined with color Doppler ultrasound (CDUS), diagnostic accuracy is further enhanced. Studies have reported sensitivities as high as 93.4% with this approach (11). In recent years, the development of artificial intelligence (AI)-assisted diagnostic technologies has markedly improved the standardization of ultrasound image acquisition, particularly in view recognition (12, 13). The application of deep learning algorithms has also enabled automated segmentation of key cardiac structures, providing strong support for precise diagnosis (14). Moreover, with advanced tools such as fetalHQ, continuous ultrasound monitoring allows for the quantification of cardiac morphological changes, facilitating dynamic assessment of fetal cardiac function (15, 16).

Current research indicates that the prenatal detection rate of complex CHD is significantly higher than that of simple CHD (17–21). Conditions such as atrial septal defects, single ventricle, hypoplastic left heart syndrome, transposition of the great arteries, tetralogy of Fallot, and double outlet right ventricle show higher detection sensitivity, whereas anomalies like pulmonary venous return abnormalities and aortic coarctation are less easily identified, consistent with the findings of this study. In this study, 3,386 pregnant women underwent fetal echocardiography, allowing for systematic evaluation of fetal cardiac anomalies and identification of various types of CHD. Based on anatomical classification, nine major types of cardiac defects were observed, with septal defects and isolated valvular abnormalities being the most common. Additionally, conditions such as hypoplastic left heart syndrome, right ventricular outflow tract anomalies, and rarer malformations like situs inversus, single atrium, and single ventricle were also identified.

Despite its essential role in detecting fetal cardiac defects, prenatal ultrasound has limitations in determining the etiology of CHD and in comprehensively assessing disease prognosis. Genetic testing can identify a wide range of genetic variants, including single nucleotide polymorphisms (SNPs), microdeletions/microduplications, and insertions/deletions, which are important genetic contributors to CHD. These variants involve hundreds of genes related to cardiac development, such as transcription factors, signaling molecules, and structural proteins (22–27). By establishing associations between specific variants and CHD subtypes (e.g., ventricular septal defect, tetralogy of Fallot), genetic testing significantly improves diagnostic precision (28–30).

On the basis of imaging findings, genetic testing provides crucial complementary information. It not only helps clarify genetic etiologies that cannot be identified by ultrasound or MRI, but also optimizes the overall diagnostic workflow. It increases the detection rate of low-frequency or rare variants, reduces false-negative and false-positive results, and is particularly valuable in high-risk families or specific CHD subtypes (31, 32). Moreover, genetic testing plays a key role in optimizing genetic counseling and risk assessment. Test results can be used to predict disease prognosis, evaluate recurrence risk, and provide personalized reproductive advice for families (33). For example, the identification of pathogenic CNVs or *de novo* mutations can help parents understand the likelihood of disease in offspring and assist in decision-making regarding pregnancy continuation or early intervention (34). Genetic testing can also reveal genetic links between CHD and other developmental abnormalities (e.g., neural tube defects, placental dysfunction), supporting more comprehensive health assessment and long-term management (35).

Genetic testing technologies have been validated in clinical practice, especially for isolated or non-isolated CHD cases (36). Furthermore, the integration of multi-omics approaches (e.g., DNA methylation analysis, AI-assisted analysis) and non-invasive methods (e.g., cfDNA analysis) continues to evolve, offering new avenues for early diagnosis and mechanistic research (37, 38). At the clinical management level, positive genetic results can guide perinatal care, such as enhancing fetal growth monitoring, optimizing delivery timing, and formulating individualized treatment plans when necessary (39). Identifying the genetic etiology also aids in predicting postoperative risks, planning interventions, and improving treatment outcomes.

In this study, 73.6% of fetuses with confirmed CHD were successfully delivered, and follow-up showed that most newborns were in good health, with no signs of neurodevelopmental disorders or other significant abnormalities. However, 61 fetuses with CHD were terminated due to complex CHD and genetic syndromes. It is important to note that these termination decisions were based on the known poor prognosis associated with complex CHD and genetic syndromes, rather than the diagnosis of CHD alone. Such conditions are often accompanied by significant neonatal morbidity and mortality, as well as long-term health issues, all of which influenced the decision-making process.

In summary, fetal ultrasound plays a vital role in CHD screening, but its limitations underscore the necessity of integrating genetic testing to achieve a higher level of prenatal diagnosis and genetic counseling. This combined strategy not only enhances diagnostic comprehensiveness and accuracy, but also provides a solid foundation for personalized medicine and public health prevention. Moreover, with the standardization of testing protocols and the integration of artificial intelligence technologies, prenatal management of CHD will become more precise and efficient. Therefore, incorporating genetic testing into comprehensive fetal genetic analysis has become a key approach to optimizing prenatal diagnosis and genetic counseling for CHD.

### 4.2 CHD and chromosomal karyotype analysis

The present study has revealed that the detection rate of chromosomal abnormalities via karyotype analysis in fetuses with CHD is significantly higher than that in fetuses without CHD. Specifically, the detection rates increase progressively from simple CHD to complex CHD and further to CHD with extracardiac anomalies, highlighting the significant diagnostic value of karyotype analysis in identifying such complex cases. Particularly in high-risk CHD cases with extracardiac anomalies, the genetic information provided by karyotype analysis is crucial for the early diagnosis and intervention of CHD.

Congenital heart disease is a multifactorial disorder involving maternal, environmental, and genetic factors (40). Genetic factors play a pivotal role in the pathogenesis of CHD, encompassing monogenic diseases, chromosomal abnormalities, and polygenic conditions (41). Chromosomal abnormalities account for approximately 20% of CHD cases, including numerical anomalies (such as aneuploidy) and structural anomalies (such as deletions, duplications, and translocations) (42). These genetic variations can lead to significant clinical manifestations, affecting not only cardiac structure but also other organ systems. Previous studies have shown that CHD is often associated with chromosomal abnormalities, gene mutations, or genetic syndromes, which can lead to severe comorbidities and poor prognosis (43). In particular, CHD cases with extracardiac anomalies should be further evaluated for additional systemic abnormalities, and fetuses with such anomalies should be tested for genetic syndromes and chromosomal abnormalities. Therefore, for fetuses diagnosed with CHD or pregnancies with significant risk factors, chromosomal and genetic testing through amniocentesis or cordocentesis is recommended.

In this study, 697 pregnant women were included, of whom 629 underwent traditional karyotype analysis. The results showed that trisomy 21 and trisomy 18 were the most common chromosomal abnormalities, with aneuploidy being more prevalent in complex CHD cases (44). However, trisomy 21 was not the most common aneuploidy in this study, which may be related to the widespread early screening for Down syndrome among pregnant women in China. Studies have shown that approximately 50% of individuals with trisomy 21 may have varying degrees of cardiac malformations, such as atrial septal defect (ASD), ventricular septal defect (VSD), and atrioventricular septal defect (45). Severe chromosomal abnormalities can lead to intrauterine fetal death. Common autosomal dominant genetic syndromes, such as DiGeorge syndrome, Holt-Oram syndrome, and Alagille syndrome, are usually associated with cardiac malformations, and conotruncal defects are associated with 22q11 microdeletion (46).

In summary, karyotype analysis has demonstrated significant diagnostic efficacy in identifying chromosomal abnormalities in CHD cases, particularly in those with extracardiac anomalies. This highlights the importance of incorporating karyotype analysis into the diagnostic workflow for high-risk CHD cases.

### 4.3 CHD and CMA

Chromosomal microdeletions and microduplications—such as 22q11.2 deletion syndrome and Williams–Beuren syndrome—are well-established causes of CHD (32). In this study, CMA identified pathogenic CNVs in 5.28% (34/644) of all CHD fetuses, and—crucially—delivered an incremental diagnostic yield over karyotyping in the same cohort. Among the 588 CHD cases with a normal karyotype, CMA detected 4.42% (26/588) additional pathogenic or likely pathogenic CNVs that were missed by conventional cytogenetics. This incremental yield rose to 7.69% (5/65) in complex CHD and 7.69% (2/26) in CHD with extracardiac anomalies, compared with 4.88% (27/553) in simple CHD. These data demonstrate that, even when karyotype analysis is normal, CMA uncovers clinically relevant submicroscopic variants, reinforcing its value as a second-tier test for refining risk assessment and guiding perinatal management.

These findings suggest that different CHD subtypes may have distinct pathogenic mechanisms, with complex CHD and CHD with extracardiac anomalies having a stronger genetic basis. Moreover, the study revealed that in CHD cases with normal karyotype results, CMA significantly increased the detection rate of pathogenic CNVs. This indicates that CMA has higher sensitivity than traditional karyotype analysis, enabling the identification of smaller chromosomal abnormalities such as microdeletions and microduplications, which are often missed by conventional karyotype analysis. These results highlight the significant diagnostic value of CMA in providing critical genetic information for early diagnosis and intervention, especially in high-risk CHD cases. The higher detection rates in complex CHD and CHD with extracardiac anomalies further underscore the importance of integrating CMA as a complementary tool to traditional karyotype analysis, particularly in cases with additional systemic anomalies.

In recent years, the rapid development of genomic technologies has revealed a large number of gene variations associated with CHD. These variations not only interfere with the normal development of the heart but may also cause extracardiac complications, thereby significantly affecting the prognosis of newborns. CMA, as an important detection method, has achieved molecular karyotype analysis through various array platforms. Compared with traditional chromosomal karyotype analysis, CMA has higher sensitivity and has been widely used in the clinical diagnosis of unexplained chromosomal abnormalities, developmental delay, autism, and various congenital malformations (47). In 2014, the Chinese expert consensus recommended CMA testing for fetuses with cardiac structural abnormalities detected by prenatal ultrasound (48). The American College of Obstetricians and Gynecologists (ACOG) and the Society for Maternal-Fetal Medicine (SMFM) also recommended the use of CMA in prenatal diagnosis of fetal structural abnormalities (49).

Connor et al. (50) reported a detection rate of 7.4% for pathogenic CNVs in 121 CHD cases, while another study (51) reported a rate of 12.0% in 514 CHD cases. In comparison, our study identified pathogenic CNVs in 5.3% of 644 CHD cases. These differences may be related to the characteristics of the study population, differences in detection methods, and sample size. The most common pathogenic CNV identified in our study was the 22q11.2 deletion, consistent with findings by Farwell et al. (52). Farwell et al. also confirmed a significant association between conotruncal defects (CTD) and *22q11.2* deletion syndrome. In our study, 10 CHD cases with *22q11.2* deletion were identified, with 9 diagnosed with CTD, further emphasizing the significance of this deletion in CHD.

Current studies have shown that CMA testing can increase the detection rate of rare genetic diseases in all pregnant women undergoing prenatal invasive testing, without considering cost factors, such as DiGeorge syndrome and Williams-Beuren syndrome (53, 54). As more pathogenic CNVs are confirmed, the positive rate of CMA is expected to increase, thereby promoting a broader understanding of the genetic risks of CHD and its associated extracardiac abnormalities (55–57).

In our study, 34 pathogenic CNVs were identified in fetuses with CHD, including *22q11.2* deletion/duplication, as well as variations in regions such as *1q21.1, 15q11.2-q13.1*, and *16p11.2*. Notably, *15q11.2* deletion is associated with Angelman syndrome and Prader-Willi syndrome (58, 59). Shaffer et al. (60) also confirmed that CMA testing can increase the detection rate of pathogenic CNVs (10.6%), especially for variants smaller than 10 Mb. Additionally, our study identified CNVs associated with rare syndromes such as Wolf-Hirschhorn syndrome and Miller-Dieker syndrome, further supporting the significance of CNVs in the pathogenesis of CHD (61).

Despite the significant increase in CNV detection rates through CMA, clinically uninformative CNVs (VOUS) still account for a part of the results (1.40%). The classification of VOUS depends on gene content, dosage sensitivity, and functional impact (62), and their pathogenicity needs to be further clarified through functional studies and family validation (63). Among the 9 VOUS in our study, 3 were possibly associated with CHD but require further verification (64–67). Although these CNVs did not meet current ACMG criteria for pathogenicity, their potential contribution to the cardiac phenotype cannot be definitively excluded. The clinical significance of VOUS depends on gene content, inheritance pattern, and functional evidence, making interpretation challenging. To refine classification and improve genetic counseling, we recommend trio-based parental CMA analysis for all fetuses with VOUS. With the integration of next-generation sequencing (NGS) and whole-exome sequencing (WES), more precise CNV interpretation may be achieved in the future (68).

In the field of prenatal diagnosis, conventional karyotyping has long served as the primary method for identifying chromosomal structural rearrangements, with a resolution capacity of approximately 5–10 megabases (Mb). However, CMA significantly enhances our diagnostic capabilities by detecting CNVs across a broader range from tens of kilobases to several megabases, thus offering much higher resolution than traditional karyotyping (69). This is precisely why CMA has become the preferred method for detecting CNVs in fetuses with normal karyotypes and CHD. Our study, which identified 34 pathogenic CNVs, underscored the high-resolution capability of CMA. The majority of these CNVs (22 out of 34, approximately 64.7%) were smaller than 5 Mb. Notably, only 12 out of 34 CNVs (approximately 35.3%) exceeded 5 Mb, highlighting CMA’s capacity to detect submicroscopic deletions and duplications that traditional methods might miss. However, when we stratified these findings by CHD subtype—simple, complex, and those with extracardiac anomalies—we observed that CNV segment size alone does not significantly differentiate among these types of CHD. The distribution of CNV sizes, categorized into segments smaller than 5 Mb and those 5 Mb or larger, did not show a statistically significant difference among the groups . Similar results were reported by Ye F et al. (70), who found no statistically significant differences in CNV size distribution frequencies between isolated and non-isolated CHD groups (*p* > 0.05), suggesting that CNV size distribution may be similar across different CHD subtypes. This indicates that the pathogenicity of CNVs is more dependent on the genomic content and dosage sensitivity of the genes involved, rather than just the length of the segment. In conclusion, in prenatal counseling, equal attention should be given to “genomic content” as well as “segment size.” By comprehensively considering both factors, we can more accurately assess phenotypic risks. This balanced approach is crucial for providing personalized and precise genetic advice, ensuring that management strategies are tailored to the specific genomic characteristics of each case.

Our study identified 27 pathogenic CNVs in 553 fetuses classified as simple CHD, including *22q11.2* deletions, *22q11.2* duplications, and other pathogenic CNVs involving regions such as *1q21.1, 15q11.2-q13.1*, and *16p11.2*. Among these, *22q11.2* duplications and deletions, as well as *15q11.2* deletions, are often associated with rare syndromes such as Angelman syndrome and Prader-Willi syndrome (71, 72). Additionally, our study revealed that CMA detected 7 CNVs related to chromosomal syndromes in the control group without ultrasound abnormalities, most of which were associated with varying degrees of postnatal developmental delay. Previous studies have identified *GATA4*, *NKX2*, and *TBX5* as contributing to the diagnosis of monogenic CHD, while *BMP4* and *CRELD1* have also been confirmed to be closely related to the occurrence of fetal CHD (73). Our study further discovered through CMA that *NF1, HNF1B*, and *MAP3K20* are associated with the pathogenesis of fetal CHD. Moreover, the variability in cardiac phenotypes detected by CMA indirectly indicates its ability to reveal small microdeletions and duplications that traditional karyotype analysis cannot detect.

Recent prospective series have reinforced the incremental value of combining CMA and exome sequencing (ES) in fetuses with CHD. In a nationwide cohort of 360 unselected CHD fetuses, CMA alone identified clinically significant CNVs in 16.7%, and ES provided an additional 6.7% diagnostic yield among those with normal CMA results, resulting in an overall 23.3% positive rate (74). Notably, non-isolated CHD showed a significantly higher detection rate (38.6%) than isolated CHD (18.8%). Similarly, in 168 fetuses with isolated ventricular septal defect (VSD), CMA revealed pathogenic CNVs in 4.2%, with perimembranous VSD carrying a markedly higher risk than muscular VSD (75). Population-based data further demonstrate that after a normal non-invasive prenatal screening (NIPS) result, the residual risk for clinically significant CMA findings remains 2.0%–2.8% in isolated CHD and 4.5%–5.9% in non-isolated CHD—substantially higher than in pregnancies without structural anomalies (76). These contemporary studies underscore that CMA—and ES when CMA is negative—should be offered to all pregnant women carrying fetuses with CHD, regardless of NIPS results or apparent isolation of the cardiac defect, to maximise diagnostic accuracy and optimise perinatal management.

In the prenatal setting, VOUS introduce pronounced counseling challenges because of their inherent ambiguity, which complicates risk communication and pregnancy management. The molecular interpretation is complex; clinical teams cannot provide clear prognoses when a VOUS is identified in clinically relevant genes, and approximately 5% of prenatal cases require extensive counseling to address both medical uncertainty and parental anxiety (77). At the decision-making level, pregnancies carrying inherited VOUS are usually continued, whereas those with *de novo* VOUS are more frequently terminated, illustrating how variant origin critically shapes parental choices and highlighting the delicate balance between scientific uncertainty, ethical considerations, and family values (78). Addressing these dilemmas necessitates ongoing, multidisciplinary dialogue—explaining evolving ACMG criteria, delivering personalized recommendations, and integrating data from antenatal and post-natal teams—so that genetic counselors can translate complex genomic findings into actionable, comprehensible information and support truly informed decisions (79, 80).

In our cohort, although VOUS constituted only 1.40% of all chromosomal microarray findings, they posed significant counseling difficulties. Among VOUS with parental data, 42.85% were reclassified through trio-based CMA, underscoring the value of familial analysis in alleviating parental anxiety and refining recurrence-risk estimates. Consequently, we now routinely offer trio-CMA and structured pre-test counseling whenever a VOUS is reported. A recent study demonstrated that trio-based whole-exome sequencing or Sanger sequencing in fetuses with CHD can unequivocally distinguish *de novo* from inherited variants (81). Similarly, in a series of 21 recurrent fetal malformations, parent-of-origin testing after prenatal ultrasound identified 18 inherited variants and three *de novo* events, further emphasizing the pivotal role of trio analysis in clarifying VOUS pathogenicity (82).

### 4.4 Synergistic role of imaging and genetics

In the prenatal diagnosis of CHD, fetal echocardiography, chromosomal karyotyping, and CMA demonstrate strong complementarity, collectively enhancing the detection rate of genetic etiologies and improving the accuracy of clinical decision-making. Fetal echocardiography serves as the primary screening tool and is the preferred method for detecting CHD and other structural anomalies (70, 83, 84). Karyotyping is mainly used to identify chromosomal numerical abnormalities and large structural variants, while CMA is more effective in detecting microdeletion/microduplication syndromes (85, 86). The combined use of karyotyping and CMA significantly improves the detection rate of chromosomal abnormalities in CHD. One study reported that the detection rate using karyotyping alone was 8.81% in pregnant women aged 35–39 and 18.18% in those aged ≥40, while the combination with CMA further increased the detection rate (87). Another study involving 427 fetuses with CHD showed that the abnormal detection rate of CMA (28.2%) was higher than that of karyotyping (19.0%) (88). The combined application of both techniques yielded a 4.08% higher detection rate than karyotyping alone and an 8% higher rate than CMA alone (57). In advanced maternal age (AMA) pregnancies, especially when accompanied by other high-risk factors, the combined use of karyotyping and CMA can effectively reduce the birth rate of fetuses with chromosomal abnormalities (89).

Current evidence suggests that when fetal ultrasound indicates structural cardiac abnormalities (e.g., ventricular septal defect, great artery anomalies) or extracardiac anomalies, further genetic testing is warranted (90). Karyotyping can rapidly identify numerical chromosomal abnormalities (e.g., trisomies) and large structural variants, and shows higher sensitivity especially in CHD cases with additional ultrasound anomalies (91, 92). CMA complements karyotyping by detecting submicroscopic chromosomal abnormalities such as microdeletions and microduplications, including the 22q11.2 deletion syndrome, thereby significantly improving the detection rate of pathogenic variants by 30%–50% compared to karyotyping alone (86). Notably, CMA can also identify potential abnormalities in isolated CHD cases (36). The combined use of karyotyping and CMA can increase the overall detection rate of genetic abnormalities in CHD fetuses to approximately 30%, with CMA contributing an additional 15%–20% of diagnostic information in karyotype-negative cases (93, 94). Moreover, the presence of extracardiac anomalies indicates a higher risk of chromosomal abnormalities, further enhancing the diagnostic value of CMA (95, 96). Identifying the underlying genetic etiology not only aids in predicting fetal neurodevelopmental outcomes but also guides reproductive decision-making and perinatal management, such as further fetal cardiac or brain MRI assessment for secondary lesions (97, 98). When CMA identifies variants of uncertain significance (VOUS), familial analysis is recommended, and karyotyping can assist in excluding macroscopic chromosomal abnormalities (28, 99, 100). Therefore, a recommended diagnostic workflow for fetal cardiac anomalies is as follows: after initial ultrasound screening identifies abnormalities, karyotyping should be prioritized to rapidly exclude common aneuploidies; subsequently, regardless of karyotype results, CMA should be performed to comprehensively detect submicroscopic structural variants. This integrated approach enables a more efficient and accurate genetic evaluation of fetal cardiac malformations.

In summary, the prenatal diagnosis of CHD is optimally achieved through the complementary integration of three core modalities: high-resolution fetal echocardiography, conventional karyotyping, and CMA. Echocardiography delivers precise anatomical delineation and guides the timing of invasive testing; karyotyping remains the gold standard for detecting aneuploidy and balanced chromosomal rearrangements; and CMA, with its kilobase-level resolution, uncovers submicroscopic CNVs and yields an incremental 4%–7% diagnostic yield in fetuses with normal karyotypes. While targeted next-generation approaches such as exome sequencing may be reserved for selected cases where CMA is non-diagnostic, the synergistic use of these three primary techniques maximizes diagnostic accuracy, expedites clinical decision-making, and provides comprehensive support for perinatal management and parental counseling.

## 5 Limitations and future research directions

This study has several limitations. First, due to resource constraints and clinical practice conditions, it was not feasible to perform postnatal echocardiography in all neonates, especially those prenatally diagnosed as healthy. Future studies should consider incorporating postnatal follow-up to validate prenatal diagnostic findings, which would further enhance the accuracy of diagnostic methods. Second, genetic abnormality detection is highly dependent on current technologies, such as CMA, which may fail to identify certain types of genetic mutations. The interpretation of CNVs and VOUS remains challenging and requires further validation. Third, differences in follow-up duration among participants may affect the assessment of long-term outcomes. Future studies should focus on larger and more diverse cohorts to enhance the reliability of genetic findings. Advanced genetic testing technologies, such as next-generation sequencing (NGS), should be employed to identify a broader spectrum of genetic variants associated with CHD. Multidisciplinary collaboration and long-term follow-up studies are needed to better understand the clinical significance of genetic findings and to improve patient outcomes. Further investigation into the functional mechanisms underlying identified genetic abnormalities is also essential for advancing our understanding of CHD etiology and developing targeted interventions.

## 6 Conclusion

Echocardiography remains the cornerstone for the prenatal detection of fetal heart malformations. When combined with karyotyping and CMA, this integrated approach achieves maximal detection of both macroscopic and submicroscopic genomic alterations—particularly in complex cardiac malformations or when extracardiac anomalies coexist—thereby delivering timely, comprehensive genetic information to guide early intervention and tailored perinatal counseling.

## Data Availability

The original contributions presented in this study are included in this article/supplementary material, further inquiries can be directed to the corresponding authors.
